# Why are we still living in the past? Sri Lanka needs urgent and timely reforms of its archaic mental health laws

**DOI:** 10.1192/bji.2022.26

**Published:** 2023-02

**Authors:** Aruni Hapangama, Jayan Mendis, K.A.L.A. Kuruppuarachchi

**Affiliations:** 1 FRANZCP, Professor in Psychiatry, Department of Psychiatry, Faculty of Medicine, University of Kelaniya, Ragama, Sri Lanka. Email: ahapangama@kln.ac.lk; 2 MDpsych, Senior Lecturer, Department of Psychiatry, General Sir John Kotelawala Defence University, Ratmalana, Sri Lanka; 3 FRCPsych, Cadre Chair and Senior Professor of Psychiatry, Department of Psychiatry, Faculty of Medicine, University of Kelaniya, Ragama, Sri Lanka

**Keywords:** Mental health act, human rights, Sri Lanka, South Asia, mental diseases

## Abstract

Mental health legislation protects the rights of people with mental illnesses. However, despite major social, political and cultural changes, Sri Lankan mental health services still operate on laws enacted mostly during the British rule more than a century ago, in the pre-psychotropics era, and focusing more on the detention of people with mental illnesses than on their treatment. It is high time all stakeholders made efforts for the much-awaited new Mental Health Act to pass through parliament urgently to meet the needs and protect the rights of patients, their caregivers and service providers.

People with mental illnesses face stigma, discrimination, marginalisation and violation of their rights; furthermore, owing to the nature of symptoms some have impaired decision-making capacity, which may result in refusal to accept treatment.^1,2^

In 2017 Sri Lanka, with its population of more than 21 million, was reported to have 2800.2 disability-adjusted life years due to mental illnesses per 100 000 population;^3^ the rate of suicides per 100 000 population was 14.6.^4^ However, Sri Lanka not only lacks enough trained clinicians (e.g. there were 0.52 psychiatrists per 100 000 population in 2017) to meet this disease burden but it also does not have up-to-date laws to govern them to provide care for people with mental illnesses in the least restrictive and least intrusive manner.^2^

Sri Lanka, which is proud of its free health services and vastly improving healthcare indices, still operates on mental health laws that were first formulated in 1873, when the country was a colony of the UK, whereas some of the other South Asian countries, such as India and Pakistan, who also inherited their mental health laws from the UK, have enacted new laws (in 2017 and 2001 respectively) in concordance with the United Nations Convention on the Rights of Persons with Disabilities.^5–8^ Sri Lanka lags far behind its South Asian neighbours with its ‘new Mental Health Act’ being in draft status since the early 2000s.^9^

Sri Lanka's Lunacy Ordinance of 1873 has undergone several amendments, the last of which was back in 1956, when it was renamed the Mental Diseases Ordinance (also known as the Mental Diseases Act).^5,10^ This act abolished the word ‘lunacy’ and introduced the words ‘mental disease’.^10^ It is worth highlighting that during the process of getting this act passed through the Parliament of Ceylon (as Sri Lanka was then known), the Cabinet Minister of Health at that time, E. A. Nugawela, reported the difficulties in getting a patient treated in or admitted to a psychiatric hospital, difficulties that we can confidently report unfortunately persist and prevail almost 70 years later.^11^

Sri Lanka has gone through many social, cultural, political and economic upheavals since 1956: the three-decade-long ethnic conflict, the 2004 Boxing Day tsunami, the Easter Sunday bombings of 2019, the ongoing COVID-19 pandemic and the currently prevailing economic crisis in the country. Over the same period, globally many recommendations have been put forward to protect the rights of people with disabilities.^5–8^ Furthermore, mental health services in Sri Lanka have been growing, with almost all the country's administrative districts now being served by a consultant psychiatrist and most districts having at least one acute in-patient psychiatry unit.

## Problems with Mental Diseases Ordinance of 1956

The Mental Diseases Ordinance of 1956 has four types of hospital admission: voluntary, temporary, emergency and admissions through district courts.^10^ The Ordinance sanctions and stipulates that all these admissions should be solely to ‘a mental hospital’ (although there was only one mental hospital at that time, located in a suburb in the Colombo district and now known as the National Institute of Mental Health, NIMH). It does not mention psychiatric units or wards in general hospitals.^5,10^

However, since 1956, acute in-patient psychiatry units have been built in all the general hospitals and at least some of the country's district hospitals and most of these units also provide out-patient clinics based in the hospitals and a few satellite clinics in remote areas. Therefore, despite having a serving consultant psychiatrist, beds and other resources in these hospitals or services located outside Colombo, patients cannot be admitted involuntary to any such unit or reviewed in a clinic if one is to follow the existing legislation.^5,10^ However, the reality is that for more than half a century patients with mental illnesses who lack the capacity to give consent are indeed admitted to and are being cared for in regional acute in-patient psychiatric facilities under the common law in the best interests of the patient but in breach of the Mental Diseases Ordinance.^10^ The patients admitted to such units may undergo seclusion and restraining procedures as well as receive treatment, including electroconvulsive therapy (ECT) following a second opinion, but none of these units, services or procedures are under the purview of the Mental Diseases Ordinance of 1956.^10^

Under the Ordinance, one form of admission is through the court system, where the allegedly mentally ill person is remanded in custody in a state prison despite not having done anything illegal until they appear before the courts and then generally that person remains in the state prison for several weeks until the procedure is completed and logistics are arranged to transfer them to the NIMH, which may be hundreds of kilometres from their place of residence as well as from the courts making such an order.^10^ What we have noticed first hand is that during such a procedure these people spend more time as ‘prisoners’ on remand without being assessed or treated than as patients at the NIMH. Such outdated policies and procedures without doubt increase stigma, violate human rights and are traumatic not only for the patient but also for their caregivers and care providers. Such traumatic procedures also discourage patients from ever re-presenting to any mental health service provider to receive care if they relapse. We need to be highly mindful that these laws were introduced in an era when none of the therapies that are used effectively now, such as psychotropics and modernised versions of ECT, were available, leading to a very high possibility that admission to the mental hospital was probably for life and with little hope of discharge before the death of the person, whereas now the mean duration of an in-patient stay in a general hospital psychiatry unit is around 1–2 weeks.^12^

In addition to the admission procedures, the Ordinance hardly addresses procedures for seclusion and restraint (chemical and or physical), which may have to be used if a patient is aggressive.^10^ The current law does not include guidelines regarding seclusion or restraint reviews or breaking of seclusion/restraint.^5,10^

Other areas of concern are the appeals procedure against admission and the requirement that the treatment or discharge of those admitted by the courts must go through the courts again, which is cumbersome and time-consuming and may prolong the time the patient remains at the NIMH.^5,10^ Furthermore, some patients lack insight and capacity and may not have the monetary or other resources to follow these tedious processes.

In addition, even though the Mental Diseases Ordinance touches on community treatment it does not describe the procedure explicitly and we believe the new Mental Health Act should describe the community management of those with mental illnesses, including community treatment orders, appeal processes, revocation or further upholding in very clear terms.

Other areas that the current legislation does not pay much attention to are admission of prison inmates, rehabilitation, housing and occupation, and disposal of property of patients who are detained under the Mental Diseases Ordinance as well as their capacity to give consent, probably because it was written at a time when institutionalisation was considered the main mode of treatment for those with severe mental health problems.

Furthermore, the Ordinance does not address the care of patients in other healthcare settings, such as private hospitals which provide the Western model of treatment as well as settings that apply other medical practices utilised in Sri Lanka (Ayurveda, Unani, etc). Private healthcare institutions and other non-governmental medical practitioners (Western and non-Western) and traditional healers being not under the purview of the mental health legislature may have deleterious consequences for both patients and carers.

## The way forward

The draft Mental Health Act of Sri Lanka, which has been in a draft stage for more than a decade, seems to address some of the issues we have raised here.^9^ However, since this draft was formulated, many sociocultural changes have occurred globally and locally which may influence how the Mental Health Act may be put into practice. These include restricted access of patients and their caregivers to mental health services owing to the COVID-19 pandemic. Utilising modern technology such as video/teleconferencing to review patients who are under the Mental Health Act but cannot been seen in person should be incorporated into the draft.^13,14^

In addition, the new Mental Health Act should reflect changes that have been made to other legislation in Sri Lanka, such as the penal code, and poisons, opium and dangerous drugs ordinances, as well as recent changes to the mental health laws of other countries.

Furthermore, it is high time that service providers and policymakers realise that the Mental Health Act is not just for providing treatment to people with mental illnesses in institutions in the least restrictive and intrusive manner, but it also provides a legal framework for addressing issues such as treating people with mental disorders in the community, their education, housing, employment as well as their integration while protecting their rights.

Getting this important piece of legislature through Parliament will give hope to other countries in the region, which are still struggling to formulate their own mental health acts.

## Data Availability

Data availability is not applicable to this article as no new data were created or analysed in this study.
